# White matter integrity as a predictor of response to treatment in first episode psychosis

**DOI:** 10.1093/brain/awt310

**Published:** 2013-11-16

**Authors:** Tiago Reis Marques, Heather Taylor, Chris Chaddock, Flavio Dell’Acqua, Rowena Handley, A. A. T. Simone Reinders, Valeria Mondelli, Stefania Bonaccorso, Marta DiForti, Andrew Simmons, Anthony S. David, Robin M. Murray, Carmine M. Pariante, Shitij Kapur, Paola Dazzan

**Affiliations:** 1 Department of Psychosis Studies, Institute of Psychiatry, King’s College London, UK; 2 Department of Neuroimaging, Institute of Psychiatry, King's College London, UK; 3 Department of Psychological Medicine, Institute of Psychiatry, King’s College London, UK; 4 National Institute for Health Research (NIHR) Mental Health Biomedical Research Centre at South London and Maudsley NHS Foundation Trust and King’s College London, UK

**Keywords:** antipsychotic drugs, psychosis, diffusion tensor imaging, white matter, schizophrenia

## Abstract

The integrity of brain white matter connections is central to a patient’s ability to respond to pharmacological interventions. This study tested this hypothesis using a specific measure of white matter integrity, and examining its relationship to treatment response using a prospective design in patients within their first episode of psychosis. Diffusion tensor imaging data were acquired in 63 patients with first episode psychosis and 52 healthy control subjects (baseline). Response was assessed after 12 weeks and patients were classified as responders or non-responders according to treatment outcome. At this second time-point, they also underwent a second diffusion tensor imaging scan. Tract-based spatial statistics were used to assess fractional anisotropy as a marker of white matter integrity. At baseline, non-responders showed lower fractional anisotropy than both responders and healthy control subjects (*P < *0.05; family-wise error-corrected), mainly in the uncinate, cingulum and corpus callosum, whereas responders were indistinguishable from healthy control subjects. After 12 weeks, there was an increase in fractional anisotropy in both responders and non-responders, positively correlated with antipsychotic exposure. This represents one of the largest, controlled investigations of white matter integrity and response to antipsychotic treatment early in psychosis. These data, together with earlier findings on cortical grey matter, suggest that grey and white matter integrity at the start of treatment is an important moderator of response to antipsychotics. These findings can inform patient stratification to anticipate care needs, and raise the possibility that antipsychotics may restore white matter integrity as part of the therapeutic response.

## Introduction

Response to treatment in first episode of psychosis is heterogeneous, with approximately 55% of patients responding to antipsychotics in the first 12 months (Boter *et al.*, 2009). The early discrimination between responders and non-responders is therefore of crucial importance, as it can reduce disability, healthcare costs, and eventually improve long-term outcome (Correll *et al.*, 2003; Ascher-Svanum *et al.*, 2008). With psychiatric neuroimaging moving towards approaches that may deliver clinically useful outcomes (Borgwardt and Fusar-Poli, 2012), attention has shifted to the identification of neuroimaging markers as potential early predictors of response. These could lead to a step forward in understanding not only the pathophysiology of the disorder, but most importantly, the neural basis of treatment response.

Most studies on neuroanatomical markers of response have investigated the role of grey matter (Bodnar *et al.*, 2012), whereas little attention has been paid to white matter. This is surprising as loss of white matter integrity has been suggested as a key component of psychotic disorders (McGuire and Frith, 1996). In fact, reductions of fractional anisotropy, a marker of white matter microstructural integrity, are already apparent at the first psychotic episode (Pérez-Iglesias *et al.*, 2010), before antipsychotic treatment (Cheung *et al.*, 2008), and even in individuals at ultra-high risk of developing psychosis (Carletti *et al.*, 2012). Nevertheless, to date, only a handful of studies have examined the association between white matter and response to treatment, with inconsistent findings (Mitelman *et al.*, 2006, 2009; Garver *et al.*, 2008; Luck *et al.*, 2011). Two cross-sectional studies, suggested an association between less extensive fractional anisotropy reductions and subsequent good clinical outcome, in the whole-brain and along tracts such as the uncinate and the superior longitudinal fasciculus (Mitelman *et al.*, 2006; Luck *et al.*, 2011). However, these studies did not perform a second evaluation of white matter at follow-up. Follow-up imaging studies can provide more information than cross-sectional studies, as they allow the identification of subtle within-subject anatomical changes and provide a true measure of the anatomical change over time that may relate to treatment outcome. The only two published longitudinal studies have been conducted in chronic patients, and they have reported conflicting results (Garver *et al.*, 2008; Mitelman *et al.*, 2009). The first study, in a small sample, showed that patients with chronic schizophrenia who responded to antipsychotics (*n = *8), as assessed 28 days after the brain imaging, had reduced white matter microstructural integrity compared with non-responders (*n = *5), who were in turn no different from control subjects (Garver *et al.*, 2008). In contrast, the second study, in a sample of chronic patients, assessed response 4 years after the brain imaging, and found that non-responders (*n = *26) had lower frontal and parietal fractional anisotropy compared with responders (*n = *23) (Mitelman *et al.*, 2009). Therefore, possibly because of the small samples and the inclusion of chronic patients, it is difficult to disentangle which brain white matter changes may predict response to treatment. Importantly, recent animal studies indicate that antipsychotics may have a protective effect on white matter (Bartzokis, 2012; Zhang *et al.*, 2012). Therefore, any longitudinal change in fractional anisotropy may be, at least in part, attributable to antipsychotics. These questions can be best addressed by investigating patients with first episode psychosis, where treatment has occurred for only a short time, and the additional effect of illness duration on brain white matter is less likely to be present.

In this study, we evaluated a large sample (*n = *63) of patients with first episode psychosis, at baseline and again at 12 weeks follow-up. To our knowledge, this is the first longitudinal diffusion tensor imaging study in first episode psychosis. To evaluate the relationship between white matter integrity and treatment response, we used a whole-brain skeleton-based technique (tract-based spatial statistics) (Smith *et al.*, 2006). We hypothesized that: (i) patients would show a decrease in white matter microstructural integrity when compared with control subjects; (ii) patients’ ability to respond to treatment would be associated with more marked alterations in white matter microstructural integrity at baseline; and (iii) antipsychotic treatment would be correlated with an improvement in white matter microstructural integrity at follow-up.

## Materials and methods

### Samples

Patients with first episode psychosis were recruited from the South London and Maudsley Foundation Trust. Healthy control subjects were recruited from the same catchment area. Healthy control subjects were administered the Psychosis Screening Questionnaire (Bebbington and Nayani, 1995), and excluded if they reported any psychotic symptom or a history of psychotic illness.

Exclusion criteria included: history of head trauma or injury with loss of consciousness >1 h; organic psychosis; learning disabilities; or lack of English fluency. Ethical permission was obtained from the Institute of Psychiatry Ethics Committee. After complete description of the study to the subjects, written informed consent was obtained.

### Assessments

All patients underwent two clinical and MRI assessments: the first as soon as possible after their first contact with services, and the second 12 weeks after. This interval was chosen because of the clinical recommendation that antipsychotics should be continued for 6–8 weeks before switching to a different medication (Taylor *et al.*, 2007); hence, a 12-week interval would provide information on treatment response after at least one drug taken for an appropriate period of time. Healthy control subjects had a single neuroimaging assessment. The mean interval between scans was 89 days (±25.4 days). A total of 63 patients and 52 healthy control subjects underwent an MRI scan at baseline (Table 1); 53 patients were re-assessed at follow-up, as 10 patients could not be traced. Forty-eight patients agreed to undergo a second MRI scan: of these, six MRI scans could not be used because of movement artefacts, leaving 42 MRI scans for inclusion in the longitudinal neuroimaging analysis. To rule out potential systematic biases, we compared clinical and socio-demographic characteristics of patients who did and did not complete the second MRI, and found no significant differences. There were no differences between groups in the proportion of antipsychotic-naive subjects at baseline, nor in exposure to mood stabilizers, antidepressants or benzodiazepines (data not shown).

**Table 1:** Demographic and clinical characteristics of responders, non-responders and healthy control subjects

	Responders (*n = *30)	Non-responders (*n = *33)	Control subjects (*n = *52)	Test statistic
Age at scan (mean years, SD)	27.7 (7.1)	27.7 (9.2)	25.1 (6.5)	df (113); *F* = 0.6; *P = *n.s.
Sex (male/female)	18/12	22/11	25/27	df (2); χ^2 ^= 2.9; *P = *n.s.
Handedness: right-handed (%)	89	94	82	df (2); χ^2 ^= 1.9; *P = *n.s.
Education (mean years, SD)	13.9 (2.9)	12.8 (2.5)	14.9 (3.1)	df (85); *F* = 17; *P = *0.09
Full scale IQ (mean, SD)	95 (14)	91 (15)	103 (16)	df (87); *F* = 0.9; *P = *n.s.
Diagnosis (% of affective psychosis)	87	75	n/a	df (1); χ^2 ^= 0.34; *P = *n.s.
Schizophrenia	80	70		
Schizoaffective disorder	7	5		
Bipolar disorder	10	17		
Depressive disorder	3	8		
Use of cannabis (%)	77	70	48	df (113); *F* = 4.0; *P = *0.19
Duration of untreated illness (duration of untreated psychosis) (mean days, SD)	116 (182)	152 (200)	n/a	df (55); t = 0.6; *P = *n.s.
PANSS total score, baseline (mean, SD)	54.5 (13.0)	62.7 (13.5)	n/a	df (61); t = 2.3; *P = *0.06
PANSS total score, follow-up (mean, SD)	41.3 (5.7)	58.7 (10.8)	n/a	df (40); t = 2.5; *P = *0.001
Total antipsychotic dose at baseline (mean CPZ equivalents, SD)	9608 (14026)	9583 (11569)	n/a	df (61); t = 0.5; *P = *n.s.
Antipsychotics used (% of atypicals)	93	91	n/a	df (1); χ^2^ = 0.15; *P = *n.s.
Total antipsychotic dose at follow-up (mean CPZ equivalents, SD)	27572 (19859)	31608 (28999)	n/a	df (40); t = 0.2; *P = *n.s.
Inter-scan interval (mean days; SD)	89 (32)	90 (17)	n/a	df (40); t = 1.3; *P = *n.s.

CPZ = chlorpromazine; n.s. = *P*-value not significant; *P*-value presented if *P < *0.1.

#### Clinical assessment

ICD-10 diagnoses were formulated by psychiatrists using OPCRIT+ (McGuffin *et al.*, 1991), with good inter-rater reliability (kappa = 0.9). Psychotic symptoms were evaluated, at baseline and follow-up on the day of MRI using the Positive and Negative Syndrome Scale (PANSS; Kay *et al.*, 1987). This scale was also used to evaluate treatment response as the primary outcome measure, as required by the Remission criteria of the Schizophrenia Working Group Consensus (Andreasen *et al.*, 2005).

These criteria identify an absolute threshold in severity of symptoms that should be reached for clinical improvement. We preferred this methodology to symptom change cut-offs, which are arbitrary, may be affected by variability in baseline symptom severity and are not intuitively understood by clinicians.

According to these criteria, patients were defined as ‘responders’ if, at 12-weeks, they had a final score of mild or less on eight defined PANSS items. For those 10 patients who could not be re-assessed at 12 weeks, information on treatment response was obtained using the Personal and Psychiatric History Schedule (PPHS; Jablensky *et al.*, 1992), which showed substantial agreement with PANSS score criteria (kappa = 0.72). Therefore, all patients assessed at baseline were classified according to their subsequent response: 30 as responders, 33 as non-responders. Duration of untreated psychosis was quantified as the interval between first psychotic symptoms and first contact with psychiatric services. Antipsychotic doses were converted to chlorpromazine equivalents (Atkins *et al.*, 1997; Woods, 2003), and a total cumulative antipsychotic dose was calculated by summing all daily doses from the first day of treatment up to each MRI scan. Fifty-five patients were taking atypical antipsychotics, five typical and three were medication-naïve. Premorbid IQ and handedness were assessed using the Wechsler Abbreviated Scale for Intelligence (Wechsler, 1997) and the Annett Hand Preference Questionnaire, respectively (Annett 1970).

### Neuroimaging evaluation

#### Image acquisition

Data were acquired on a 3.0 T, GE Signa-HDx system running software release 14M5 with actively shielded magnetic field gradients (maximum amplitude 40 mT/m). Body coil was used for radiofrequency transmission, with 8-channel head coil for signal reception, allowing a parallel imaging (ASSET) speed-up factor of two. Each volume was acquired using a multi-slice peripherally-gated doubly refocused spin echo-echo planar imaging sequence, optimized for precise measurement of the diffusion tensor in parenchyma, from 60 contiguous near-axial slice locations with isotropic (2.4 × 2.4 × 2.4 mm) voxels. Echo time was 104.5 ms and repetition time varied between 12–20 R–R intervals. Maximum diffusion-weighting was 1300 s/mm^2^, and at each slice location, four images were acquired with no diffusion gradients applied, together with 32 diffusion-weighted images with gradient directions uniformly distributed in space. An inhouse automated analysis technique assessed the quality of echo planar imaging data (Simmons *et al.*, 1999). Acquisition was gated to the cardiac cycle using a peripheral gating device placed on the subjects’ forefingers.

#### Image processing

Diffusion data were processed using ExploreDTI. Data were first preprocessed correcting for eddy current distortions and head motion. For each subject the b-matrix was then re-oriented to provide a more accurate estimate of tensor orientations. The diffusion-tensor was estimated using a non-linear least square approach, with fractional anisotropy calculated from the diffusion-tensor. Voxel-wise statistical analysis of fractional anisotropy was carried out using TBSS v1.2 (tract-based spatial statistics) (http://www.fmrib.ox.ac.uk/fsl/tbss/) (Smith, 2002; Smith *et al.*, 2004, 2006) to compare groups of diffusion-weighted measures. First, fractional anisotropy images were registered to standard MNI space using the non-linear registration tool in FSL (FNIRT) to the JHU-ICMB-DTI-81 atlas. A voxel-wise average of all subjects was used to create a study-specific mean fractional anisotropy image, which was then ‘skeletonized’ to create a mean fractional anisotropy skeleton, representing the centres of all white matter tracts. To exclude low anisotropic regions in the skeleton, a fractional anisotropy threshold of 0.3 was applied. Each subjects aligned fractional anisotropy maps were then projected onto this skeleton and results fed into voxel-wise cross-subject statistics.

#### Diffusion tensor imaging tractography

To investigate whether differences in white matter microstructural integrity were related to changes in white matter volume, a tractography analysis of the tracts identified as most affected in the whole-brain comparisons between responders and non-responders was performed using TrackVis, with placement of regions of interest described elsewhere (Catani *et al.*, 2002; Catani and Thiebaut de Schotten, 2008). For each tract the number of streamlines was calculated to obtain proxy measurements of tract volume.

### Statistical analysis

Whole-brain statistical analyses were performed using Randomise v2.1 (FSL). Longitudinal changes in fractional anisotropy were assessed by subtracting the follow-up skeletonized fractional anisotropy image from each individual’s baseline image. Statistical comparisons used the threshold-free cluster enhancement (TFCE), using a non-parametric permutation test, in which group membership was permuted 5000 times to generate a null distribution for each contrast. Significant clusters were identified where voxel-wise *P*-values were corrected for multiple comparisons [*P < *0.05, family-wise error (FWE) corrected]. Age, gender and handedness were de-meaned before analysis and used as covariates of no-interest within the voxel-based analysis. Because of differences in the duration of untreated psychosis between groups, duration of untreated psychosis was calculated and added as a covariate after logarithmic transformation. To localize significant voxel effects, contrast maps were subdivided according to the 48 regions of the JHU-ICBM-DTI-81 white matter atlas. This allowed quantification of the number of significant voxels within each regional mask, and spatial identification of the peak voxel. We then extracted the individual’s mean fractional anisotropy value for clusters identified as significantly different between responders and non-responders at baseline, and for the same clusters at follow-up, to quantify progressive fractional anisotropy change in these regions. For these clusters we also estimated the values of other white matter diffusion tensor imaging parameters: mean diffusivity (a composite measure as fractional anisotropy, which increases with the loss of structural barriers that normally restrict water diffusion); axial diffusivity (which is more specific to axonal integrity and can be considered an index of axonal damage); and perpendicular diffusivity, suggested to be a marker of reduction in the myelin content, hence representing an index of axonal demyelination) (Song *et al.*, 2002, 2003).

Socio-demographic and clinical characteristics were analysed using one-way ANOVA for continuous variables, Mann-Whitney for skewed distributions and chi-square for categorical variables. Repeated measures ANOVA were used for the analysis of extracted fractional anisotropy values and tractography volumes. Analyses were conducted using SPSS v.18.0.

## Results

### Baseline differences in white matter microstructural integrity

#### Patients versus healthy control subjects

A total of 63 patients and 52 healthy control subjects underwent an MRI scan at baseline, and their demographic and clinical characteristics are presented in Table 1.

**Figure 1:**
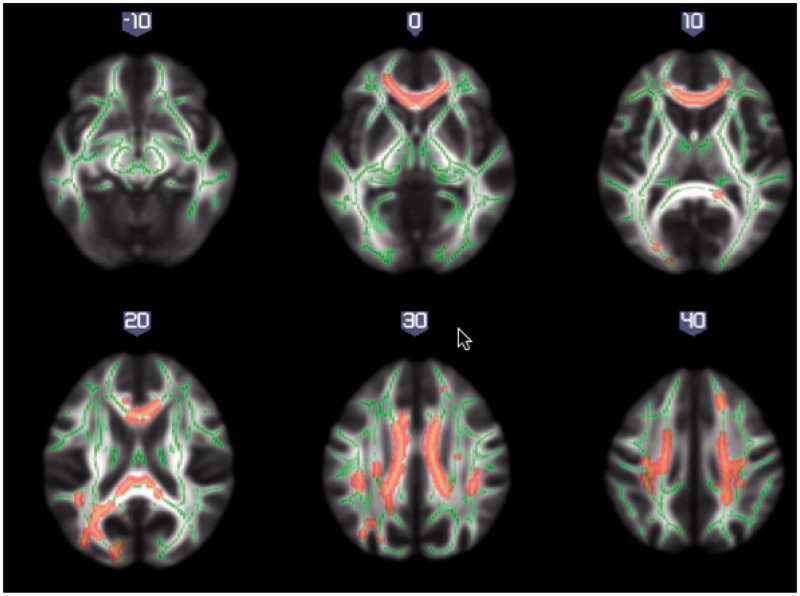
White matter maps showing significantly decreased fractional anisotropy in all patients when compared to healthy control subjects, at baseline (*P < *0.05, FWE-corrected). Background image corresponds to the mean fractional anisotropy image in standard MNI152 brain space (radiological view). Fractional anisotropy white matter skeleton is represented by green voxels. Red–yellow voxels represent regions in which the fractional anisotropy was significantly lower in the patient group relative to the healthy control group.

We first compared the white matter maps of the whole first episode psychosis patient group with the maps of the healthy controls at baseline. This comparison revealed areas of reduced fractional anisotropy values across the brain in first episode psychotic patients (*P < *0.05; FWE-corrected), with the most affected tracts including corpus callosum, superior longitudinal fasciculus, corona radiata and left cingulum and thalamic radiation (Fig. 1) (for details of regions see Supplementary Table 1).

#### Responders versus non-responders

**Figure 2:**
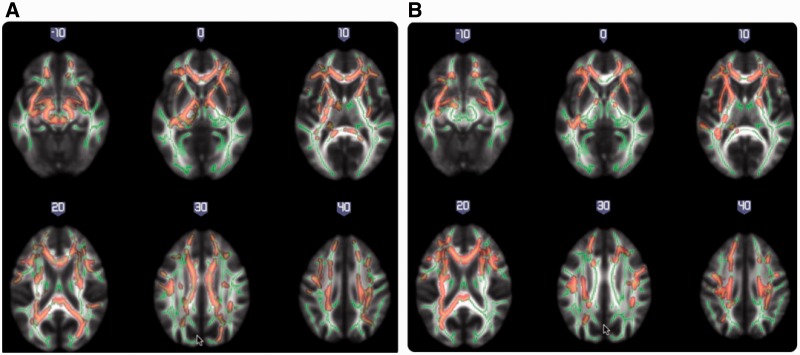
White matter maps showing significantly decreased fractional anisotropy in non-responders when compared with responders, at baseline (*P < *0.05, FWE-corrected) (**A**); and at 12-weeks follow-up (*P < *0.05, FWE-corrected) (**B**). Background image corresponds to the mean fractional anisotropy image in standard MNI152 brain space (radiological view). Fractional anisotropy white matter skeleton is represented by green voxels. Red–yellow voxels represent regions in which the fractional anisotropy was significantly lower in the non-responder group relative to the responder group.

We then examined differences in white matter microstructural integrity at baseline between responders and non-responders, and found that non-responders had significantly lower fractional anisotropy across multiple brain regions when compared to responders (*P < *0.05; FWE-corrected) (Fig. 2A). The most affected tracts comprised associative fibres, including the uncinate, stria terminalis and superior frontal-occipital tract; commissural fibres such as the corpus callosum; and several projections fibres, such as the internal and external capsule and corona radiata. The tracts with highest percentage of significant voxels were those interconnecting frontal and temporal cortices (uncinate and fornix) (Table 2). We re-ran this analysis including total baseline PANSS scores as a covariate, together with follow-up length, and the results remained significant, with an overlap in the affected regions. There were no regions in which non-responders showed significantly higher fractional anisotropy than responders.

**Table 2:** White matter regions of fractional anisotropy reduction between non-responders and responders at baseline

JHU white matter atlas region	Significant cluster size (*n* voxels)	% Region significant	*t*-statistic	MNI coordinates of peak voxel (mm)		
				*x*	*y*	*z*
Uncinate						
Left	70	91%	3.38	−33	−6	−14
Right	59	86%	2.24	35	−4	−14
Fornix						
Left stria terminalis	270	76%	2.98	−34	−11	−15
Right stria terminalis	11	4%	2.64	34	−11	−15
Corticospinal						
Left	203	75%	1.92	9	−29	−23
Right	135	51%	3.02	10	−27	−27
Cerebral peduncle						
Left	436	72%	2.35	−9	−29	−20
Right	360	59%	3.40	10	−28	−19
External capsule						
Right	781	67%	2.48	36	−7	−13
Left	790	61%	4.36	−35	−8	−13
Internal capsule						
Left posterior limb	575	66%	2.64	−20	−14	0
Right anterior limb	480	58%	3.24	10	1	1
Left retrolenticular limb	372	47%	2.44	−24	−19	0
Left anterior limb	361	43%	3.00	−13	−1	4
Corpus callosum						
Body	1985	63%	2.70	−13	17	24
Genu	1039	60%	3.55	−15	32	14
Splenium	1145	46%	2.13	1	−31	21
Corona radiata						
Right anterior	1005	63%	3.26	27	28	10
Left anterior	942	56%	3.75	−15	33	15
Left posterior	364	48%	1.27	−21	−44	35
Left superior	578	42%	2.36	−22	−25	36
Right superior	591	42%	3.21	21	−16	36
Right posterior	153	20%	2.67	30	−60	19
Cerebellar peduncle						
Left superior	99	48%	2.16	−5	−30	−21
Right superior	90	36%	2.46	7	−31	−16
Left inferior	66	33%	1.97	−13	−45	−32
Middle	753	33%	3.19	−23	−46	−36
Pontine cross tract	98	25%	2.35	−2	−30	−30
Medial lemniscus						
Left	61	24%	2.13	−4	−38	−37
Right	30	12%	2.10	6	−34	−25
Superior frontal occipital						
Right	18	23%	1.52	23	2	19
Left	9	10%	1.45	−21	5	19

JHU = Johns Hopkins University; % Region significant was obtained by dividing the number of significant voxels within each regional mask by the total number of voxels in that mask.

#### Non-responders versus healthy control subjects

Similarly, non-responders also had a significantly lower fractional anisotropy across multiple white matter regions when compared to healthy controls (*P < *0.05; FWE-corrected). The most affected tracts included associative tracts, such as the left uncinate, cingulum and superior longitudinal fasciculus, and commissural tracts such as the corpus callosum (for details of the regions see Supplementary Fig. 1 and Supplementary Table 2) There were no white matter regions where controls had significantly lower fractional anisotropy values than non-responders.

#### Responders versus healthy control subjects

Interestingly, when we compared the baseline white matter maps of the responders to those of the healthy controls, we found no differences in fractional anisotropy values across the brain between these two groups.

### Differences in white matter microstructural integrity at 12-weeks follow-up and longitudinal changes

We compared cross-sectionally the white matter microstructural integrity at follow-up, in those 42 subjects with two usable scans: 20 responders and 22 non-responders. Non-responders continued to display lower fractional anisotropy compared with responders, mostly in the same brain tracts where they differed at baseline, including the uncinate, corona radiata, fornix, external and internal capsule and corpus callosum, although the differences appeared less widespread (*P < *0.05; FWE-corrected) (Fig. 2B and Table 3). In addition, the cingulum and the superior and inferior longitudinal fasciculus were also affected (*P < *0.05; FWE-corrected).

**Table 3:** White matter regions of fractional anisotropy reduction between non-responders and responders at 12-weeks follow-up

JHU white matter atlas region	Significant cluster size (*n* voxels)	% Region significant	*t*-statistic	MNI coordinates of peak voxel (mm)		
				*x*	*y*	*z*
**Uncinate**						
Left	58	81%	2.48	−34	−6	−14
Right	65	96%	1.88	36	−3	−16
**External capsule**						
Left	831	64%	4.61	−33	−6	−13
Right	341	29%	2.55	36	−7	−13
**Corona radiata**						
Left anterior	1014	59%	2.70	−15	36	−1
Right anterior	978	59%	3.49	23	19	18
Left superior	760	54%	3.56	−22	−25	35
Right superior	292	21%	3.47	23	−19	38
Left posterior	406	53%	2.19	−26	−30	27
Right posterior	21	3%	1.76	19	−26	35
**Fornix**						
Left stria terminalis	202	58%	2.55	−28	−26	−8
Right stria terminalis	10	3%	2.63	34	−11	−15
**Internal capsule**						
Left anterior limb	446	53%	2.51	−17	6	9
Right anterior limb	327	39%	3.13	21	4	15
Left retrolenticular limb	286	36%	2.01	−26	−24	8
Left posterior limb	160	18%	1.42	−22	−9	11
Right posterior limb	88	10%	2.25	20	−4	10
**Corpus callosum**						
Genu	806	46%	2.91	−13	33	−3
Body	725	23%	3.01	15	17	25
Splenium	377	15%	2.10	−23	−54	17
**Superior frontal occipital**						
Left	34	35%	2.25	−21	14	19
Right	26	32%	1.27	23	2	19
**Superior longitudinal fasciculus**						
Left	331	24%	2.36	−35	−25	35
Right	367	24%	1.89	38	−39	28
**Thalamic radiation**						
Left posterior	207	19%	1.73	−28	−53	18
**Inferior longitudinal fasciculus**						
Left sagittal stratum	38	8%	2.54	−35	−11	−14
Right sagittal stratum	7	1%	2.19	36	−11	−14
**Cingulum**						
Right	25	7%	2.30	10	17	26

JHU = Johns Hopkins University.

We then evaluated longitudinal within-group changes over the 12 weeks. A whole-brain analysis of within-group, and Group × Time interaction for fractional anisotropy change did not identify any significant difference between responders and non-responders. We then explored whether the regions identified at baseline as being significantly different between responders and non-responders at baseline had changed over time, and we found an overall increase in fractional anisotropy over the 12-weeks [*F*(1,42) = 16.7; *P < *0.001]. Interestingly, there was no Group (responders versus non-responders) × Time (baseline versus follow-up) interaction for these fractional anisotropy changes [*F*(1,42) = 2.53, *P = *0.12], suggesting that changes were broadly in the same direction in both groups.

To explore if changes in white matter microstructural integrity were driven by volumetric changes, we explored whether there had been longitudinal changes in the volume of the most affected tracts. However, there was no significant longitudinal change in volume in any of the tracts analysed [right uncinate, *F*(1,41) = 0.367, *P = *0.549; left uncinate, *F*(1,41) = 0.681, *P = *0.415; or fornix, *F*(1,41) = 0.808, *P = *0.374]. There was also no Group × Time interaction for white matter tracts volume (*P = *0.44 to *P = *0.78) and no significant correlations were observed between longitudinal changes in volumes and longitudinal changes in fractional anisotropy or exposure to antipsychotics.

### White matter microstructural integrity, exposure to antipsychotics and psychopathology

We further explored whether the fractional anisotropy differences we observed between responders and non-responders were accompanied by a change in other diffusion tensor imaging measures in these brain regions. Results showed that at baseline responders had significant lower mean and perpendicular diffusivity than non-responders in the white matter regions that differentiated these two groups [*F*(1,63) = 7.50, *P < *0.001; and *F*(1,63) = 17.17, *P < *0.001, respectively]. In contrast, there were no differences in axial diffusivity [*F*(1,63) = 2.374; *P = *0.1]. Furthermore, we found an overall increase in axial diffusivity [*F*(1,42) = 0.97; *P = *0.32] and a decrease in mean diffusivity and perpendicular diffusivity [*F*(1,42) = 0.1.17, *P = *0.29; and *F*(1,42) = 2.60, *P = *0.11, respectively].

**Figure 3:**
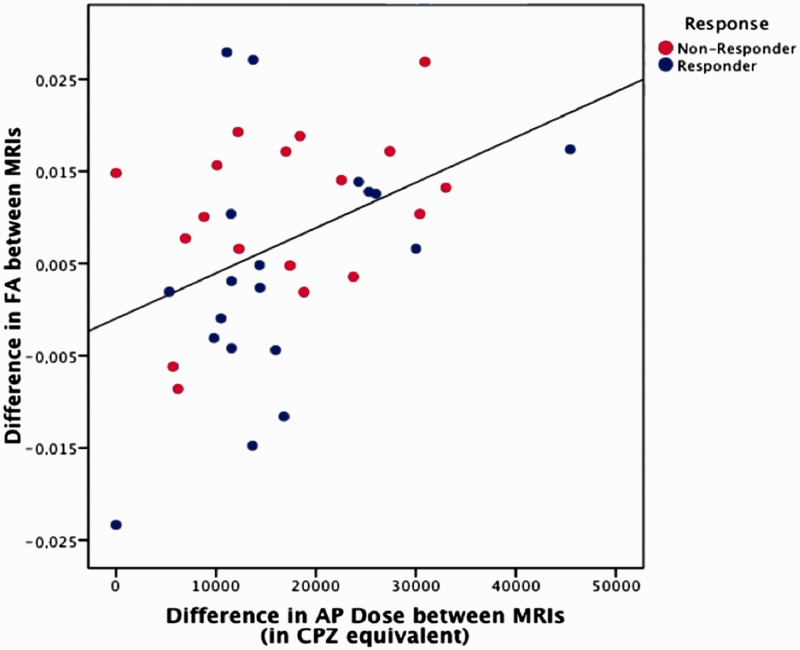
Relationship between total exposure to antipsychotic (AP) medications over the follow-up period [chlorpromazine (CPZ) equivalents] and longitudinal change in fractional anisotropy, in responders (blue) and non-responders (red).

We then explored whether differences in fractional anisotropy could be explained as an effect of antipsychotic medication. We found that, at baseline, the cumulative dose of antipsychotic was not correlated with fractional anisotropy values in the regions that discriminated responders from non-responders. However, the longitudinal change in fractional anisotropy (between baseline and follow-up scans) within these brain regions was positively correlated with the cumulative antipsychotic dose at follow-up [r(40) = 0.37, *P = *0.02], indicating that the higher the exposure to antipsychotic medication over the 12-week period, the larger the increase in fractional anisotropy (and therefore the improvement in white matter microstructural integrity) (Fig. 3). Additionally, for those same regions we also found a significant negative correlation between exposure to antipsychotic medication and perpendicular diffusivity [r(40) = −0.273, *P = *0.046], but not with mean and axial diffusivity.

Finally, in the regions that discriminated responders from non-responders at baseline, a significant negative correlation was observed between fractional anisotropy and baseline PANSS total score [r(63) = −0.273, *P = *0.046], as well as between PANSS total scores and follow-up fractional anisotropy values in these regions [r(42) = −0.581, *P < *0.001]. However, there was no correlation between change in psychotic symptoms and change in fractional anisotropy.

## Discussion

To our knowledge, this is the first longitudinal study investigating white matter microstructural changes and treatment response in first episode psychosis. In this large sample, we found that non-responders already have significantly impaired white matter microstructure at baseline, when compared with both healthy control subjects and future responders. In contrast, responders are not distinguishable from controls. Furthermore, fractional anisotropy differences between responders and non-responders remain present, albeit to a lower extent, even at follow-up. Interestingly, both patient groups show an increase in white matter microstructural integrity over time in these regions, which seems associated with the extent of exposure to antipsychotics.

Our main finding is that at the time of the first episode of illness, subsequent non-responders already show altered white matter microstructural integrity than responders. So far, results from the few existing studies assessing white matter microstructural integrity and clinical outcome have been inconsistent, possibly because of small samples, different follow-up periods and longer duration of illness. However, consistent with our findings, a cross-sectional tractography study showed that first episode patients who do not respond to 6 months of treatment have lower fractional anisotropy than responders in the superior longitudinal fasciculus and the uncinate (Luck *et al.*, 2011). The uncinate has been suggested to play an important role in the pathophysiology of psychosis and response to treatment (Van Veelen *et al.*, 2011) and indeed it was the most significantly affected tract in our study. In addition, we found substantial impairments in other tracts, such as the stria terminalis. This tract is part of the extended amygdala, and acts as a relay site within the main stress response system, the hypothalamic–pituitary–adrenal (HPA) axis. Alterations in the stress response have been linked to the pathophysiology of psychosis, as suggested by evidence of an enlarged pituitary gland and raised salivary cortisol in early stages of illness (Garner *et al.*, 2009; Mondelli *et al.*, 2010).

Interestingly, the cross-sectional comparison of the white matter maps obtained at the 12-week follow-up showed that the differences between responders and non-responders were still present, although less widespread. We found that higher PANSS scores were correlated with lower fractional anisotropy in the regions that differentiated non-responders from responders. This supports previous evidence of a negative correlation between fractional anisotropy and PANSS positive symptoms, mainly in the left uncinate and superior longitudinal fasciculus (Skelly *et al.*, 2008). The presence of this negative correlation at both baseline and follow-up suggests that this association may represent a ‘trait’ marker, related to the underlying pathophysiology, which changes very little with treatment. However, we cannot exclude that the altered white matter integrity was already present in non-responders long before illness onset, reflecting a different, possibly neurodevelopmental origin. Further support for a heterogeneous pathophysiology across patients with psychosis comes from our finding that responders show no difference in white matter integrity from controls. This finding might also help to explain inconsistencies in previous diffusion tensor imaging studies of psychosis. Although studies in chronic patients suggest a reduced fractional anisotropy when compared with control subjects (Kubicki *et al.*, 2005), studies in first episode are less consistent (White *et al.*, 2011).

Finally, these findings extend and confirm our previous data on grey matter, in a different first episode psychosis sample, showing that patients who subsequently do not experience symptom remission are significantly different from both healthy controls and those who have an episodic illness course, whereas this latter group is indistinguishable from healthy control subjects (Mourao-Miranda *et al.*, 2012). Consistently with those data, we have also found, in a sample partially overlapping with the one used in this study, that non-responders have prominent hypogyria at insular, frontal and temporal regions when compared with responders and healthy control subjects, whereas responders are not distinguishable from healthy control subjects (Palaniyappan *et al.*, 2013). Taken together, this evidence supports the combined use of white and grey matter measures as potential early neuroimaging markers for short and long-term clinical outcome, which could potentially guide treatment decisions.

Intriguingly, although there were no whole-brain significant longitudinal differences between groups, there was an increase in fractional anisotropy values in the regions that differentiated responders and non-responders at baseline, which was positively correlated with higher cumulative antipsychotic dose. Furthermore, the increase in fractional anisotropy and axonal diffusivity was accompanied by a decrease in mean and perpendicular diffusivity. This finding is difficult to interpret, because of the paucity of previous longitudinal studies. Cross-sectional studies suggest that chronic patients show more marked fractional anisotropy reductions than first episode patients, and the existing longitudinal studies also provide evidence of progressive changes in fractional anisotropy after illness onset (Mori *et al.*, 2007; Garver *et al.*, 2008; Mitelman *et al.*, 2009; Wang *et al.*, 2013). This was also evident in a recent longitudinal study in first episode schizophrenia, which identified a progressive reduction in fractional anisotropy at 6 weeks follow-up in the white matter microstructure around the bilateral anterior cingulate gyrus and the right anterior corona radiata (Wang *et al.*, 2013). It is therefore intriguing to speculate whether the increase we observed reflects a restoration of white matter microstructural integrity, promoted by some ‘protective’ factors activated by antipsychotic medications. The impact of antipsychotics on white matter remains controversial. Recently, it has been suggested that first episode patients who receive more antipsychotics show a greater reduction in white matter volume (Ho *et al.*, 2011), whereas other studies report no relationship between fractional anisotropy and dose of antipsychotic medication (White *et al.*, 2011; Wang *et al.*, 2013). In contrast, some studies suggested that antipsychotics have a pro-myelinating effect, promoting myelin repair and oligodendrocyte differentiation (Xiao *et al.*, 2008), thus improving white matter microstructural integrity (Bartzokis, 2012). Similarly, a diffusion tensor imaging animal study showed that mice that were concomitantly treated with an antipsychotic and cuprizone, a copper chelator that induces oligodendrocyte loss and demyelination, showed an attenuation of the cuprizone-induced white matter changes compared with mice receiving cuprizone alone (Chandran *et al.*, 2012). In patients, antipsychotics have been shown to increase white matter volume and intracortical myelin (Bartzokis *et al.*, 2009). However, only the aforementioned study by Garver *et al.* (2008) has used diffusion tensor imaging to evaluate patients before and after antipsychotics. This study showed a reduction in mean diffusivity over time in responders, interpreted as reflecting a partial restoration of myelin integrity. Our results extend these findings to first episode patients, using fractional anisotropy as well as other diffusion tensor measures. In our study, there was a positive correlation between fractional anisotropy and exposure to antipsychotics, together with a negative correlation between perpendicular diffusivity and exposure to these drugs. These data are important, because although fractional anisotropy can be regarded as a weighted average of the different eigenvalues, perpendicular diffusivity may be modulated by myelin quantity and has been suggested to be a marker of myelin content (Song *et al.*, 2002, 2003). Therefore, the longitudinal diffusion tensor imaging changes we observed could represent an effect of antipsychotics on white matter, potentially resulting from changes in myelin content. Finally, it is unlikely that the longitudinal fractional anisotropy change we observed was related to volumetric changes, as we found no significant difference in white matter volume over time in the most affected tracts. This suggests that a reduction in white matter volume is unlikely to have driven our findings.

Our study has a number of strengths. This is the first diffusion tensor imaging study in first episode psychosis to use a longitudinal design to explore the relationship to treatment outcome. Second, this is the largest diffusion tensor imaging study conducted to date in first episode psychosis, as previous studies used an average sample size of 30 patients. Third, our study highlights the potential relationship between white matter and antipsychotics, supporting a role for these drugs in restoring white matter microstructural integrity, while informing the neurobiological basis of treatment response. The effect of antipsychotics in white matter has been a matter of strong debate, and our study supports a potential beneficial role of these drugs on this brain functional component. Finally, this is one of the few applications of tract-based spatial statistics to first episode psychosis and the first time tract-based spatial statistics has been used to evaluate treatment response in this particular patient population. Tract-based spatial statistics offers several advantages over standard voxel-based analytic techniques, particularly relevant to longitudinal analyses, as it removes the need for spatial smoothing and minimizes the methodological pitfalls caused by misalignment and misregistration, consequently increasing the sensitivity and interpretability of findings. Also, compared to standard tractography techniques, tract-based spatial statistics is a fully automated process, which allows the whole brain to be investigated, whereas tractography, as a traditional region of interest approach, only provides information on pre-selected regions, and suffers from intrinsic operator variability.

In terms of limitation, 10 of our patients could not be contacted at follow-up, and we cannot exclude that they may have represented a different group in terms of their white matter microstructural integrity. However, their demographic and clinical characteristics were similar to the remaining sample, and this bias is therefore unlikely.

In conclusion, this work provides evidence that white matter microstructural integrity can be helpful in the early identification of patients less likely to respond to antipsychotic drugs. In combination with other neuroimaging and clinical measures, these findings could considerably help patient stratification in psychiatry, ultimately allowing individualized patient management from the time of the first presentation to services. Furthermore, by identifying underlying brain structure and connectivity as potential factors moderating antipsychotic response, it raises the question as to whether treatments that enhance and restore brain connectivity may elicit a better response to antipsychotics in those who currently do not respond to them.

## Funding

This work was supported by the National Institute for Health Research (NIHR) Mental Health Biomedical Research Centre at South London and Maudsley NHS Foundation Trust and King’s College London. The views expressed are those of the author(s) and not necessarily those of the NHS, the NIHR or the Department of Health. The study was also partially supported by a King’s College London Translational Research Grant to P. Dazzan. P. Dazzan’s research is also supported by the Psychiatry Research Trust. Tiago Reis Marques and P. Dazzan’s research are supported by NARSAD. A.A.T. Simone Reinders is supported by the Netherlands Organization for Scientific Research (NWO-VENI grant no. 451-07-009).

## Supplementary material

Supplementary material is available at *Brain* online.

Supplementary Data

## Abbreviation

PANSS: Positive and Negative Syndrome Scale
