# Supplementary material for: White matter integrity as a predictor of response to treatment in first episode psychosis
Source: Brain. 2013 Nov 16;137(1):172–82. doi: 10.1093/brain/awt310 (PMC3891445; doi:10.1093/brain/awt310)
Supplement: Supplementary Data [file supp_137_1_172__index.html]

White matter integrity as a predictor of response to treatment in first episode psychosis — Supplementary Data 

# White matter integrity as a predictor of response to treatment in first episode psychosis

## Supplementary Data

files

**Files in this Data Supplement:**

- Supplementary Data - docx file
