# Supplementary material for: White matter integrity as a predictor of response to treatment in first episode psychosis
Source: Brain. 2013 Nov 16;137(1):172–82. doi: 10.1093/brain/awt310 (PMC3891445; doi:10.1093/brain/awt310)
Supplement: Supplementary Data [file supp_awt310_brain-2013-00747-File002.docx]

**Table S1.** White Matter regions of FA reduction between First-Episode Patients and Healthy Controls at baseline.

| JHU White Matter Atlas Region | Significant cluster size (nº voxels) | % Region significant | t-statistic | MNI coordinates of peak voxel (mm) | | |
| --- | --- | --- | --- | --- | --- | --- |
|  |  |  |  | **X** | **Y** | **Z** |
| **Cingulum** |  |  |  |  |  |  |
| Left | 241 | 57% | 1.80 | -8 | -15 | 33 |
| **Corpus Callosum** |  |  |  |  |  |  |
| Body | 1519 | 48% | 2.26 | 3 | 19 | 17 |
| Genu | 1043 | 59% | 4.55 | 4 | 29 | 8 |
| Splenium | 802 | 32% | 4.04 | -17 | -35 | 29 |
| **Corona Radiata** |  |  |  |  |  |  |
| Right Anterior | 131 | 8% | 2.08 | 16 | 35 | 4 |
| Left Anterior | 96 | 6% | 2.21 | -15 | 36 | -1 |
| Left Posterior | 63 | 8% | 1.85 | -26 | -25 | 29 |
| Left Superior | 350 | 25% | 2.16 | -19 | -18 | 38 |
| Right Superior | 249 | 18% | 2.53 | 25 | -21 | 39 |
| Right Posterior | 86 | 11% | 2.66 | 20 | -40 | 34 |
| **SuperiorLongitudinal Fasciculus** |  |  |  |  |  |  |
| Right | 261 | 18% | 2.86 | 39 | -33 | 31 |
| Left | 224 | 16% | 3.63 | -37 | -34 | 30 |
| **Thalamic Radiation** |  |  |  |  |  |  |
| Left | 73 | 6% | 1.95 | -29 | -66 | 15 |

JHU = Johns Hopkins University; MNI = Montreal Neurological Institute

**Figure S1.** White matter maps showing significantly decreased FA in Non-Responders when compared to Healthy Controls, at baseline (p<0.05, FWE-corrected). Background image corresponds to the mean FA image in standard MNI152 brain space (radiological view). FA white matter skeleton is represented by green voxels. Red–yellow voxels represent regions in which the FA was significantly lower in the Non-Responder group relative to the Healthy Control group.


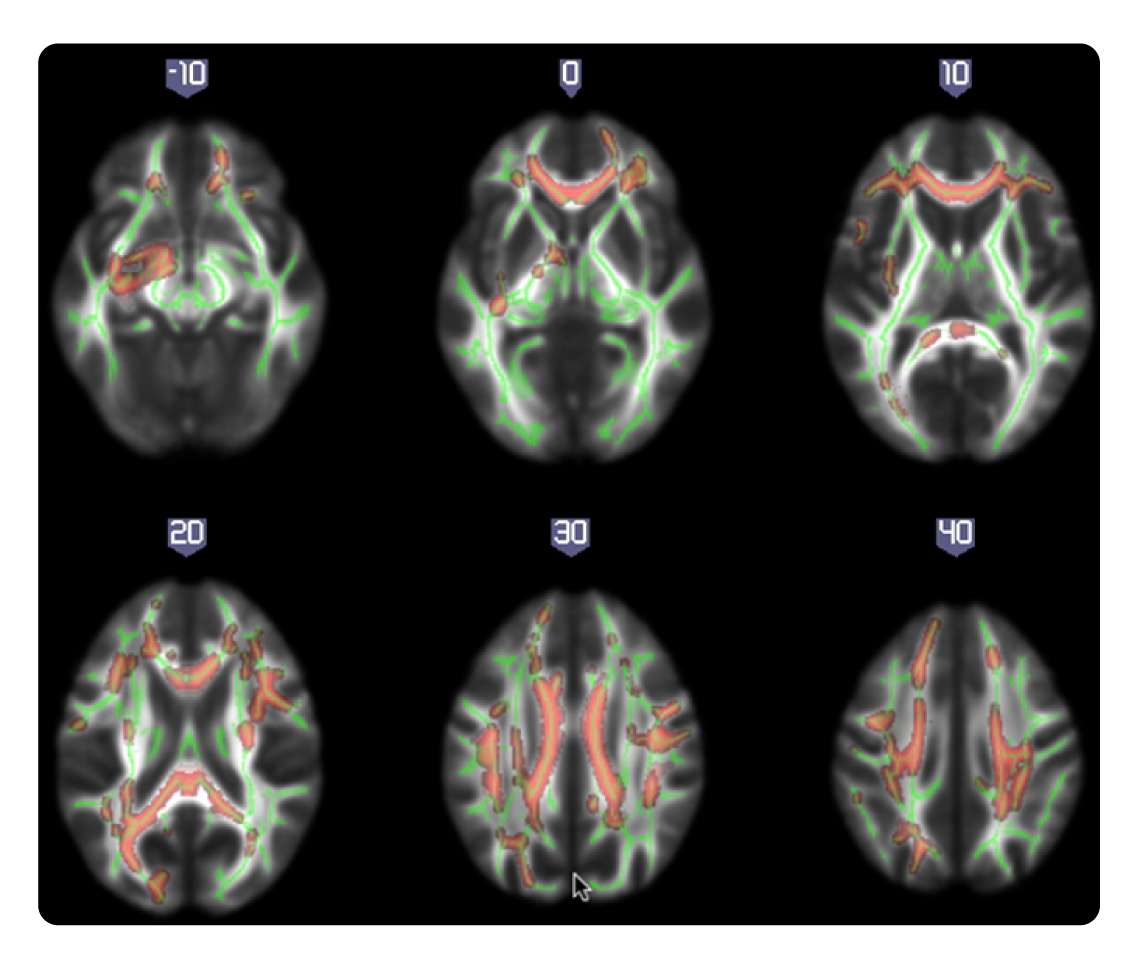


FA = Fractional Anisotropy; FWE = Family-Wise Error; MNI = Montreal Neurological Institute

**Table S2.** White Matter regions of FA reduction between Non-Responders and Healthy Controls at baseline.

| JHU White Matter Atlas Region | Significant cluster size (nº voxels) | % Region significant | t-statistic | MNI coordinates of peak voxel (mm) | | |
| --- | --- | --- | --- | --- | --- | --- |
|  |  |  |  | **X** | **Y** | **Z** |
| **Uncinate** |  |  |  |  |  |  |
| Left | 60 | 76% | 2.19 | -33 | -6 | -14 |
| **Corpus Callosum** |  |  |  |  |  |  |
| Genu | 1283 | 73% | 4.18 | -8 | 27 | -3 |
| Body | 2370 | 75% | 3.55 | 7 | -29 | 23 |
| Splenium | 1302 | 52% | 3.57 | 10 | -33 | 24 |
| **Cingulum** |  |  |  |  |  |  |
| Left | 195 | 46% | 1.66 | -9 | 16 | 26 |
| Right | 46 | 12% | 2.33 | 11 | -46 | 23 |
| **Superior Longitudinal Fasciculus** |  |  |  |  |  |  |
| Left | 451 | 33% | 1.40 | -36 | -34 | 27 |
| Right | 704 | 47% | 2.01 | 31 | -16 | 35 |
| **Corona Radiata** |  |  |  |  |  |  |
| Left Anterior | 737 | 44% | 2.79 | -14 | 34 | -3 |
| Right Anterior | 586 | 59% | 2.41 | 17 | 36 | 3 |
| Left Superior | 626 | 45% | 2.63 | -22 | -25 | 36 |
| Right Superior | 560 | 41% | 2.52 | 19 | -23 | 37 |
| Left Posterior | 236 | 32% | 1.75 | -29 | -51 | 19 |
| Right Posterior | 140 | 18% | 1.94 | 21 | -33 | 39 |
| **Fornix** |  |  |  |  |  |  |
| Left stria terminalis | 110 | 32% | 3.53 | -33 | -12 | -14 |
| **External Capsule** |  |  |  |  |  |  |
| Left | 275 | 22% | 2.02 | -33 | -6 | -13 |
| Right | 6 | 1% | 3.04 | 29 | -12 | 18 |
| **Internal Capsule** |  |  |  |  |  |  |
| Left Anterior Limb | 23 | 3% | 1.54 | -9 | -1 | -1 |
| Left Retrolenticular Limb | 79 | 10% | 2.85 | -35 | -25 | -2 |
| Left Posterior Limb | 34 | 4% | 1.66 | -12 | -6 | -4 |
| Right Posterior Limb | 47 | 5% | 2.57 | 28 | -12 | 18 |
| **Thalamic Radiation** |  |  |  |  |  |  |
| Left Posterior | 207 | 19% | 3.06 | -28 | -67 | 16 |
| Right Posterior | 28 | 2% | 3.87 | 29 | -66 | 17 |
| **Inferior Longitudinal Fasciculus** |  |  |  |  |  |  |
| Left sagittal stratum | 21 | 4% | 1.99 | -39 | -9 | -15 |

JHU = Johns Hopkins University; MNI = Montreal Neurological Institute

| **Table xxxx Volumetric white matter changes at baseline and follow-up.** | | | | | | | | |
| --- | --- | --- | --- | --- | --- | --- | --- | --- |
|  | Baseline | | 12-week  Follow-up | |  | | Group x Time Interaction | |
|  | Mean | S.D. | Mean | S.D. | Test statistic | p value | Test statistic | p value |
| Fractional Anisotropy (FA) | 0.430 | 0.170 | 0.432 | 0.148 | F(1,37) = 3.67 | .063 | F(1,37) = 0.06 | .80 |
| Mean Diffusivity (MD) | 0.000103 | 0.000532 | 0.000102 | 0.000362 | F(1,37) = 3.55 | .067 | F(1,37) = 0.36 | .54 |
| Perpendicular Diffusivity | 0.000775 | 0.000458 | 0.000778 | 0.000679 | F(1,37) = 0.03 | .55 | F(1,37) = 1.23 | .27 |
| Axial Diffusivity | 0.00155 | 0.000761 | 0.00154 | 0.000517 | F(1,37) = 2.18 | .14 | F(1,37) = 0.40 | .52 |
| **Values are mean ± standard deviation (S.D.). Significant differences are shown using asterisk (*). The level of significance for group differences was set at p=0.05** | | | | | | | | |
